# Uptitration of statin therapy in women and men: a population-based cohort study

**DOI:** 10.1093/ehjqcco/qcaf017

**Published:** 2025-04-17

**Authors:** Pauline Kiss, Alicia Uijl, Diederick E Grobbee, Monika Hollander, Elisabeth Smits, Miriam C J M Sturkenboom, Sanne A E Peters

**Affiliations:** Julius Center for Health Sciences and Primary Care, University Medical Center Utrecht, Universiteitsweg 100, 3584 CG Utrecht, The Netherlands; Julius Center for Health Sciences and Primary Care, University Medical Center Utrecht, Universiteitsweg 100, 3584 CG Utrecht, The Netherlands; Department of Cardiology, Amsterdam University Medical Centers, Amsterdam Cardiovascular Sciences, University of Amsterdam, 1105 AZ Amsterdam, The Netherlands; Division of Cardiology, Department of Medicine, Karolinska Institutet, 171 77 Stockholm, Sweden; Julius Center for Health Sciences and Primary Care, University Medical Center Utrecht, Universiteitsweg 100, 3584 CG Utrecht, The Netherlands; Julius Center for Health Sciences and Primary Care, University Medical Center Utrecht, Universiteitsweg 100, 3584 CG Utrecht, The Netherlands; Department of General Practice, Amsterdam Public Health research institute and Amsterdam Cardiovascular Sciences, Amsterdam University Medical Centres, 1007 MB Amsterdam, The Netherlands; PHARMO Institute for Drug Outcomes Research, 3528 AE Utrecht, The Netherlands; Julius Center for Health Sciences and Primary Care, University Medical Center Utrecht, Universiteitsweg 100, 3584 CG Utrecht, The Netherlands; Julius Center for Health Sciences and Primary Care, University Medical Center Utrecht, Universiteitsweg 100, 3584 CG Utrecht, The Netherlands; The George Institute for Global Health, School of Public Health, Imperial College London, London W12 7RZ, UK; The George Institute for Global Health, University of New South Wales, Sydney, NSW 2000, Australia

**Keywords:** Sex differences, Cardiovascular diseases, Statin, Electronic health records, Drug utilisation utilization research

## Abstract

**Aims:**

Statins are widely used for the prevention and management of cardiovascular disease (CVD). Women have been shown to be less likely to receive guideline-recommended dose of statins and reach target lipid levels. This study aims to examine sex differences in titration patterns of statin therapy and in the attainment of cholesterol targets in a Dutch healthcare setting.

**Methods and results:**

Data on statin dispensing was extracted from the PHARMO Data Network between 2011 and 2020. New-statin users with at least two recorded statin dispenses were included. Cox proportional hazards models were used to study the association between sex and time to first uptitration of intensity and Poisson regressions were used to estimate sex differences in the attainment of cholesterol targets within 6 and 18 months after statin initiation. We identified 68 150 new users of statin therapy (46% women) with a median age of 65 years [Q1–Q3: 57–72]. The cumulative incidence of uptitration after 3 years of follow-up was 10% in women and 12% in men. After adjustment for age, CVD and other individual characteristics, women were 28% less likely to be uptitrated compared to men (adjusted hazard ratio for women vs. men 0.72 [95% confidence interval (CI) 0.69–0.75]). The adjusted risk ratio of achieving cholesterol target levels within 6 and 18 months after statin initiation in women vs. men were 0.95 (95% CI 0.93–0.97) and 0.98 (95% CI 0.97–0.99).

**Conclusion:**

Among new statin users, women are less likely to be uptitrated compared to men and to achieve cholesterol target levels.

## Graphical Abstract

**Graphical Abstract:**
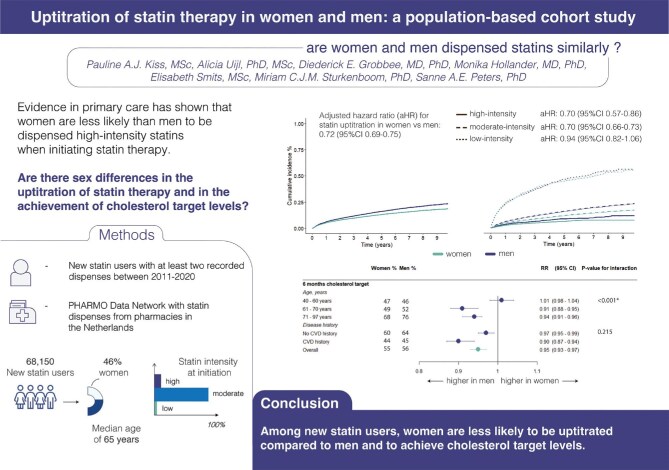

Key Learning Points **What is already known**

- Statin therapy is widely recommended in clinical guidelines to lower cholesterol levels and to prevent or treat cardiovascular diseases.
- Previous evidence showed that women are less likely to be prescribed a statin as compared to men, and, when prescribed, are given lower intensities compared to men.

**What this study adds**

- Among new statin users, women were less likely than men to be uptitrated for statin therapy, despite higher baseline cholesterol levels and after adjustment of individual characteristics and initial statin intensity.
- Women were less likely to achieve target levels of LDL-c 6 months and 18 months after the initiation of statins.

## Introduction

Cardiovascular disease (CVD) remains the leading cause of death globally, accounting for a third of all deaths in men and women annually, imposing a significant burden on healthcare systems and impacting the quality of life of millions.^1,2^ In the Netherlands, CVD, including acute myocardial infarction, stroke, or heart failure, accounted for 23% of total mortality in both women and men in 2022.^3^

Statin therapy is widely recommended in clinical guidelines to lower cholesterol levels and to prevent or treat CVD. Meta-analyses of randomized controlled trials have shown that the efficacy of statins in lowering CVD risk is similar in women and men.^4^ Hence, clinical guidelines provide similar recommendations for the use of statins in both sexes.^5^ Yet, previous studies have shown that women are less likely to be prescribed a statin as compared to men.^6–8^ Among those prescribed a statin, women are prescribed lower intensities as compared to men.^6,7,9^ Moreover, women on statins are less likely to achieve target levels of cholesterol than men,^9–11^ sometimes irrespective of the statin intensity at initiation.^9,10^

Clinical guidelines recommend to monitor blood cholesterol levels in the months following the initiation of statins, until the achievement of target levels, and, if needed, to intensify the therapy by increasing the intensity of statin or adding ezetimibe, until target levels are achieved.^5^ Whether the uptitration of statin therapy differs between women and men is not yet known.

The aim of this study is to assess whether there are sex differences in the occurrence of uptitration of statins and to assess whether there are sex differences in the achievement of cholesterol target levels within 6 and 18 months after statin initiation.

## Methods

### Source and study population

This study was conducted using electronic healthcare records (EHR) from the PHARMO Data Network. PHARMO is a population-based network of EHR databases that combines data from primary and secondary healthcare settings in the Netherlands. Data from general practitioners (GPs) (∼20% of the Dutch population), out-patient pharmacies (∼25% of the Dutch population), and hospitals (80% of hospitals in the Netherlands) are linked on a patient level through probabilistic linkage. Probabilistic record linkage creates record identifiers by using patient characteristics and comparing similar records, using likelihood estimations to determine how probable it is that a record from one linked database and a record from a second linked database refer to the same patient. PHARMO follows more than 10 million Dutch residents with an average follow-up of 12 years.^12^ PHARMO GP data has shown to be representative of the Dutch population in terms of demographic characteristics and diagnoses in primary care.^13^

In this study, individuals with linked data from the GP and out-patient pharmacy data were included if they had: (i) at least two statin dispenses between January 2011 and December 2020; (ii) a maximum 1-year gap between the first two dispenses; (iii) at least 1 year of look-back prior to index date (t0), with the index date defined as the date of the first statin dispense in the community pharmacy; and (iv) had no previous statin dispense (including statin-ezetimibe combinations) in the year before index date.

We excluded individuals that had a missing information for the statin dose at index date. We also excluded individuals with a first dispense in 2020, to ensure a minimum follow-up time of 1 year and the opportunity to have a second dispensing in that year. Individuals were followed from index date to death, loss to follow-up, or end of follow-up, whichever came first. A flow diagram of the study population and a graphical depiction of the study design can be found in Supplementary materials online, 1 and 2.

### Data extraction

The closest measurement to index date, occurring in the year prior to inclusion was extracted for each individual as baseline measurement. The following variables were extracted from the GP records at baseline: systolic blood pressure, diastolic blood pressure, body mass index (BMI), glucose, smoking, total cholesterol (TC), low-density lipoprotein (LDL-c), high-density lipoprotein (HDL-c), triglycerides (TG), creatinine, estimated glomerular filtration rate (eGFR), hemoglobin, and glycated hemoglobin (Hba1c). LDL-c measurements within 6 and 18 months after index date were also extracted and, in case of multiple measurements within the timeframe, the lowest recorded value was used per individual.

Age and socio-economic status (SES) at baseline were extracted. SES was categorized as low, middle, high, and unknown and was based on four-digit postal code and neighborhood scores from the Netherlands Institute for Social Research that account for income, education, and employment.^14^ Smoking status at baseline was extracted based on International Classification of Primary Care (ICPC) codes and free text information, and was classified into current smoking, previous smoking, and never-smoking. Medical history of CVD, coronary heart disease, stroke, heart failure, diabetes and family history of ischemic heart disease (IHD) were extracted from the GP medical records and hospital discharge data based on ICPC and International Classification of Diseases—9th and 10th revision (ICD-9, ICD-10) codes. Definitions and codes for medical history can be found in Supplementary materials online, 3.

Statin daily dose information was extracted from the dispensing records in the out-patient pharmacy data and when missing, data were retrieved from the linked prescription in the GP data, if available. When multiple statins with the same anatomical therapeutic chemical (ATC) classification were dispensed on the same day (e.g. simvastatin 20 mg and simvastatin 40 mg), the daily doses were summed (e.g. 60 mg). A frequency of one was assumed for each dispensing. Ezetimibe and statin combinations with ezetimibe were extracted from the out-patient pharmacy data. Statins were grouped into three intensities (i.e. low, moderate, and high), based on the daily dose and the ATC code, following the classification from the 2013 ACC/AHA cholesterol guideline^15^ (Supplementary material online, 4). Ezetimibe and statin combinations dispenses were considered by allocating an intensity score to each dispense (Supplementary material online, 5). The year of the first dispensed statin was extracted as year of initiation.

Data on the use of antihypertensives and antiplatelets were retrieved from the out-patient pharmacy data, using the ATC system. Drug use was defined as at least one recorded dispensed prescription in the 6 months before index date. Supplementary material online, 3 contains the codes for drug use.

### Outcome

The study outcomes were (i) first statin uptitration, defined as an increase in intensity compared to the baseline intensity, at any time following statin initiation, which could include the addition of ezetimibe, and (ii) the achievement of LDL-c target levels in the first 6 months and 18 months after statin initiation. Following Dutch guidelines, LDL-c target levels in patients with CVD ≤70 years old was 1.8 mmol/L and in patients with CVD >70 years old or individuals without a history of CVD was 2.6 mmol/L.^16^

### Data analysis

Baseline characteristics and the cumulative incidence of statin uptitration are presented by sex. Descriptive analyses were used in this study, including summary statistics and frequency counts. Multiple imputation stratified by sex for 10 imputation sets was used to impute missing data with the *mice* package in R statistical software. The model specification for the imputation can be found in Supplementary material online, 6. Results were pooled using Rubin's rules.

Cox proportional hazards models were used to estimate the adjusted hazard ratios (HRs) and 95% confidence intervals (CIs) for the association between sex and time to first uptitration. Analyses were adjusted for age, SES, CVD history, diabetes, LDL-c, smoking status, BMI, year of initiation, statin intensity at initiation, use of blood pressure-lowering drugs, use of antiplatelets, and family history of IHD. The analyses were stratified by age (i.e. 40–60, 60–70, and 70–97 years old) and statin intensity at initiation. The Cox proportional hazard assumption was verified with the Schoenfeld residuals. Strata for year of initiation was added to enhance the validity of the proportional hazards assumption.

We also investigated sex differences in statin uptitration among individuals that did not achieve LDL-c target levels in the 6 months following statin initiation (see Supplementary material online, 2b). Individuals were followed from 6 months after initiation (i.e. the landmark point) until statin uptitration, death, loss to follow-up or end of follow-up. The criterium was based on the imputed dataset and we also limited the population to the individuals with at least one statin dispensing after the landmark point. We performed Cox regressions to estimate the association between sex and time to uptitration after the landmark point. The analyses were adjusted with the same covariates as the main analysis, stratified by age and statin intensity at initiation and strata for the year of initiation.

Risk ratios (RRs) and 95% CIs for the association between sex and the achievement of LDL-c target levels at 6 and 18 months after statin initiation were calculated by Poisson regressions with robust standard errors. The analyses were adjusted with the same covariates as the Cox proportional hazard model plus in an additional sensitivity analysis also for the uptitration status within 6 and 18 months after statin initiation. Analyses were stratified by age and CVD history.

All analyses were conducted with R statistical software version 4.1.2.^17^

## Results

### Study population

The study cohort included 68 150 new statin users. Of these, 31 085 were women (46%) and the median age was 65 years [Q1–Q3: 57–72]. History of CVD was present in 31% of women and 41% of men and the mean values for LDL-c at baseline were 4.2 mmol/L [standard deviation (SD): 1.0] in women and 3.9 mmol/L (SD: 1.0) in men. Most individuals started statin therapy with a statin of moderate-intensity (87% of women and 84% of men), followed by high-intensity (8% of women and 13% of men), and low-intensity (5% of women and 3% of men) (*Table 1*).

**Table 1:** Baseline table

	Missing, *n* (%)	Overall 68 150 (100)	Women 31 085 (46)	Men 37 065 (54)
**General characteristics**				
Age [median (IQR); years]	0 (0)	65 [57–72]	66 [58–73]	64 [57–71]
SES (%)*^†^	48 (0)			
High		17 889 (26)	8018 (26)	9871 (27)
Moderate		28 550 (42)	12 991 (42)	15 559 (42)
Low		21 663 (32)	10 063 (32)	11 600 (31)
Smoking (%)*^†^	36 378 (53)			
Never		11 821 (37)	7026 (45)	4795 (30)
Previously		12 809 (40)	5298 (34)	7511 (46)
Current		7142 (23)	3276 (21)	3866 (24)
**Clinical characteristics [mean (SD)]** **^a^**				
Body mass index (kg/m^2^)*	38 657 (57)	27.8 [25.1–31.1]	27.9 [24.8–31.6]	27.7 [25.4–30.5]
Systolic blood pressure (mmHg)*	25 785 (38)	140.4 (18.8)	140.15 (19.0)	140.65 (18.5)
Diastolic blood pressure (mmHg)*	25 832 (38)	81.8 (10.5)	81.1 (10.2)	82.5 (10.7)
LDL-c (mmol/L)*	27 129 (40)	4.1 (1.0)	4.2 (1.0)	3.9 (1.0)
HDL-c (mmol/L)*	26 924 (40)	1.3 (0.4)	1.5 (0.4)	1.2 (0.3)
Total cholesterol (mmol/L)*	26 872 (39)	6.2 (1.1)	6.5 (1.1)	6.0 (1.1)
Triglycerides (mmol/L)*	27 187 (40)	1.6 [1.2–2.3]	1.6 [1.2–2.2]	1.7 [1.2–2.4]
Glucose (mmol/L)*	29 575 (43)	5.6 [5.1–6.3]	5.5 [5.1–6.2]	5.7 [5.2–6.4]
HbA1c (mmol/mol)*	55 125 (81)	44.0 [39.0–52.0]	44.0 [39.0–51.0]	44.0 [39.0–53.0]
Hemoglobin (mmol/L)*	49 043 (72)	9.0 (0.9)	8.6 (0.7)	9.3 (0.8)
Creatinine (μmol/L)*	26 647 (39)	78.0 [68.0–90.0]	70.0 [62.0–78.0]	86.0 [77.0–96.0]
eGFR (mL/min/1.73 m^2^)*	28 135 (41)	62.0 [60.0–81.0]	60.0 [60.0–79.0]	64.0 [60.0–83.0]
**Disease history (%)** **^b^**				
CVD	0 (0)	24 873 (37)	9653 (31)	15 220 (41)
CHD	0 (0)	9319 (14)	3012 (10)	6307 (17)
Stroke	0 (0)	5699 (8)	2626 (8)	3073 (8)
Heart failure	0 (0)	682 (1)	314 (1)	368 (1)
Diabetes	0 (0)	6193 (9)	2790 (9)	3403 (9)
Family history of IHD	0 (0)	3010 (4)	1194 (4)	1816 (5)
**Medications (%)^c^**				
Antihypertensives	0 (0)	45 668 (67)	21 018 (68)	24 650 (67)
Antiplatelets	0 (0)	27 641 (41)	10 852 (35)	16 789 (45)
**Intensity initiation (%)**	0 (0)			
High		7283 (11)	2472 (8)	4811 (13)
Moderate		58 112 (85)	27 109 (87)	31 003 (84)
Low		2565 (4)	1438 (5)	1127 (3)
Statin + ezetimibe		189 (0)	65 (0)	124 (0)

SD, standard deviation; LDL-c, low-density lipoprotein cholesterol; HDL-c, high-density lipoprotein cholesterol; CVD, cardiovascular disease; CHD, coronary heart disease; and IHD, ischemic heart disease.

a Refers to the last measurement in the year before statin initiation.

b Refers to any disease event occurring before statin initiation.

^c^Refers to any prescription dispensed in the 6 months prior to statin initiation.

* The variable was imputed.

† The percentage refers to complete cases. BMI, triglycerides, glucose, HbA1c, creatinine, and eGFR are expressed in median [interquartile range (IQR)].

### Sex differences in statin uptitration

**Figure 1:**
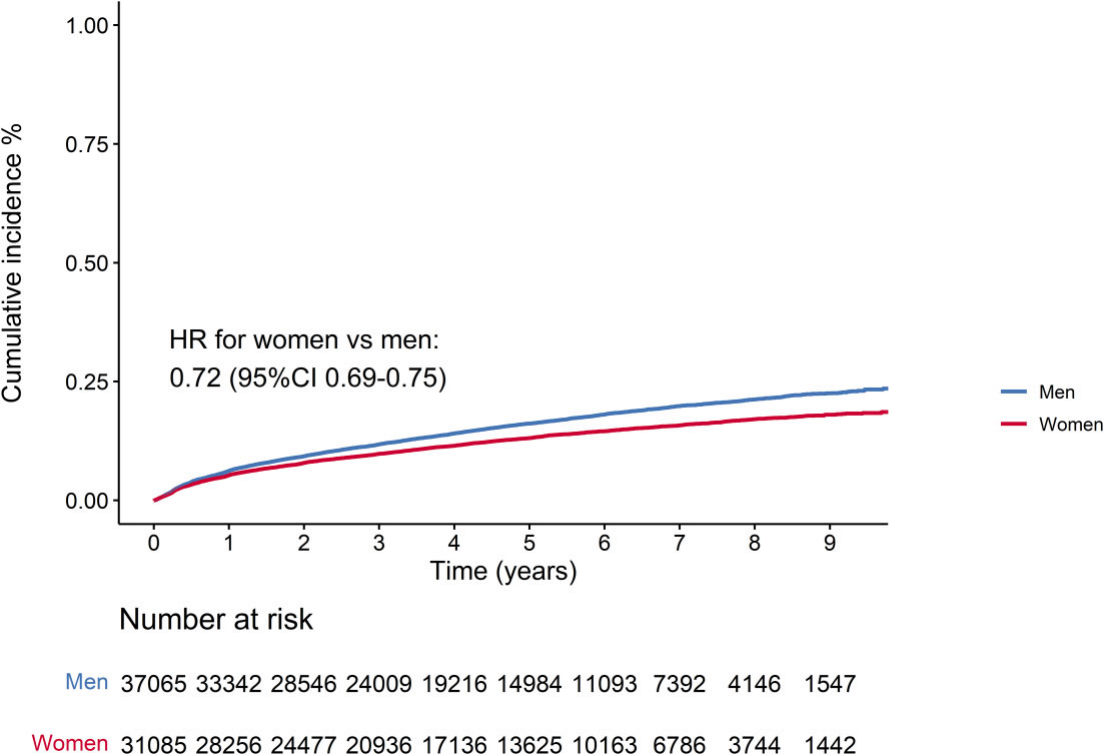
Kaplan meier curves for the time to first uptitration. HR, hazard ratio; adjusted for age, SES, CVD history, diabetes, LDL-c levels, smoking status, BMI, year of initiation, statin intensity at initiation, use of blood pressure-lowering drugs, use of antiplatelets, and family history of IHD.

**Figure 2:**
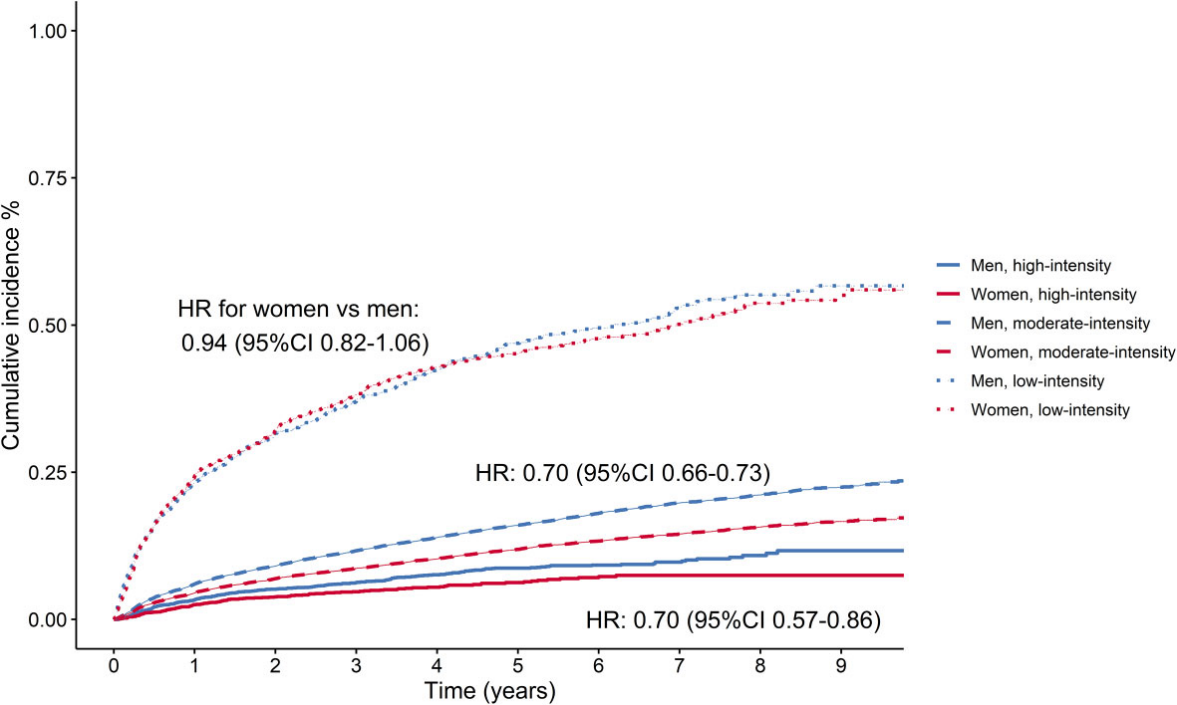
Kaplan–Meier curves for the time to first uptitration, stratified by statin intensity at initiation. HR, hazard ratio; adjusted for age, SES, CVD history, diabetes, LDL-c levels, smoking status, BMI, year of initiation, statin intensity at initiation, use of blood pressure-lowering drugs, use of antiplatelets, and family history of IHD. HR: hazard ratio and CI: confidence interval.

Over a follow-up period of 304 545 person-years, 9627 individuals were uptitrated. The median follow-up time was 4.3 years [Q1–Q3: 2.2–6.6]. The cumulative incidence of uptitration in women vs. men was 1.7% vs. 1.9% after 3 months, 3.3% vs. 3.8% after 6 months, 5.3% vs. 6.2% after 1 year, and 9.8% vs. 11.8% after 3 years of follow-up (*Figure 1*). The cumulative incidence of uptitration was the highest in individuals that initiated statin therapy with a low-intensity (cumulative incidence after 1 year in women, in men: 24%, 23%), followed with moderate-intensity (5%, 6%) and high-intensity (3%, 3%) (*Figure 2* and Supplementary material online, 7).

**Figure 3:**
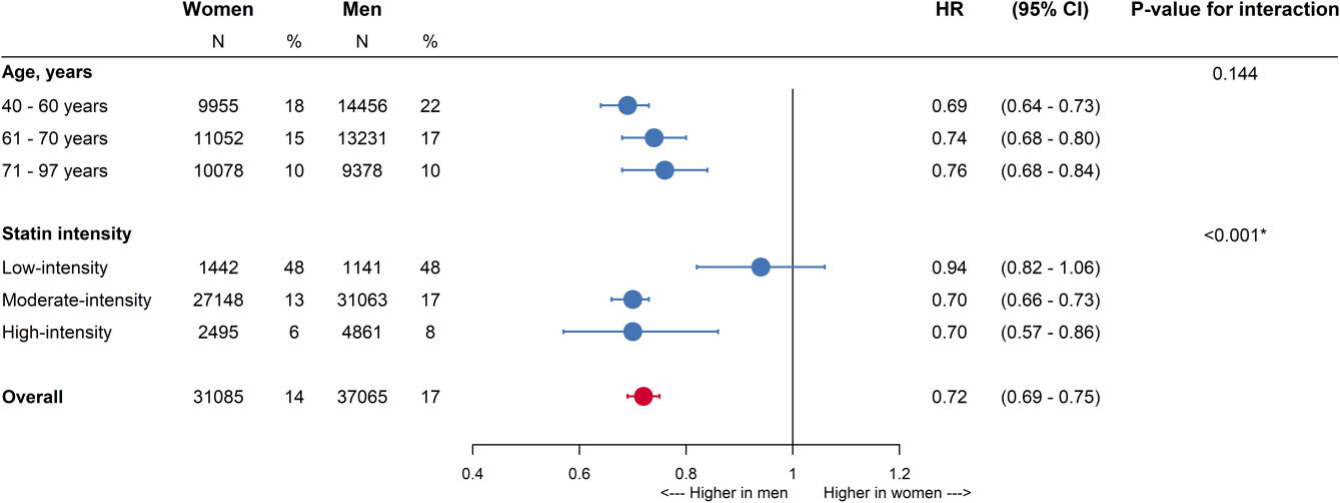
Women-to-men hazard ratios of statin uptitration—stratified by statin intensity at initiation and age category. The analyses were adjusted for age, SES, CVD history, diabetes, LDL-c levels, smoking status, BMI, year of initiation, statin intensity at initiation, use of blood pressure-lowering drugs, use of antiplatelets, and family history of IHD. The *N* refers to the number of women and men in each subgroup and the percentage refers to the percentage of women and men that were uptitrated within each subgroup. HR: hazard ratio and CI: confidence interval.

When adjusting for differences in baseline characteristics, women were 28% less likely to be uptitrated than men [HR 0.72 (95% CI 0.69–0.75)] (*Figure 3*). There was no statistically significant interaction between sex and age (*P*-value = 0.144). The women-to-men HRs for statin uptitration were 0.69 (0.64–0.73) for those 40–60 years old, 0.74 (0.68–0.80) for those 61–70 years, and 0.76 (0.68–0.84) for those 71–97 years. There were variations across subgroups of statin intensity at initiation (*P*-value for the interaction <0.001). The women-to-men HRs for statin uptitration were HR 0.94 (0.82–1.06) in individuals starting with low-intensity statins, 0.70 (0.66–0.73) in individuals starting with moderate-intensity statins, and 0.70 (0.57–0.86) in individuals starting with a high-intensity statins. Supplementary material online, *Table S8* shows the results per intensity and age combination and the results of the unadjusted analyses are shown in Supplementary material online, 9.

**Figure 4:**
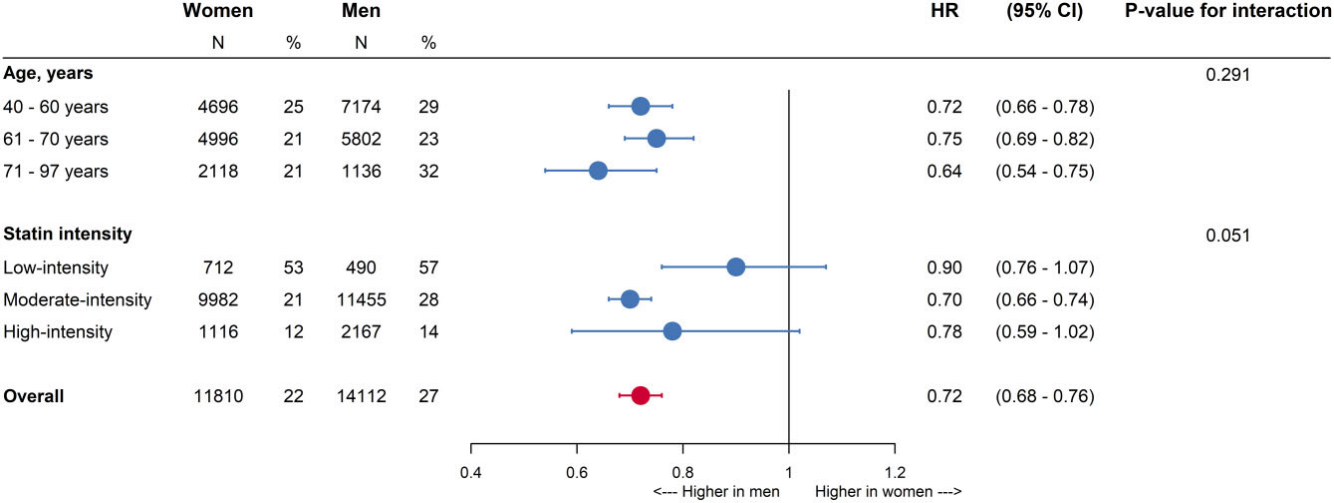
Women-to-men hazard ratios of statin uptitration among individuals without LDL-c achievement at 6 months—stratified by statin intensity at initiation and age category. The main analyses were adjusted for age, SES, CVD history, diabetes, LDL-c levels, smoking status, BMI, year of initiation, statin intensity at initiation, use of blood pressure-lowering drugs, use of antiplatelets, and family history of IHD. The *N* refers to the number of women and men in each subgroup and the percentage refers to the percentage of women and men that were uptitrated within each subgroup. HR: hazard ratio and CI: confidence interval.

A total of 25 922 (11 810 women and 14 112 men) did not achieve LDL-c target levels within 6 months after statin initiation (Supplementary material online, 2b). Women were less likely to be uptitrated compared to men [HR 0.72 (95% CI 0.68–0.76)] (*Figure 4*). Sex differences were seen across all age categories and there was no interaction between sex and age (*P*-value: 0.291). The women-to-men HRs for statin uptitration were 0.90 (0.76–1.07) for individuals with a low-intensity statin at initiation, 0.70 (0.66–0.74) for the moderate-intensity, and 0.78 (0.59–1.02) for the high-intensity (*P*-value for the interaction: 0.051).

### Sex differences in LDL-c target achievement

Overall, 54.9% (95% CI 54.7–55.0) of women and 55.8% (55.7–56.0) of men had LDL-c levels below the recommended targets within 6 months after statin initiation [median values: 2.3 mmol/L (Q1–Q3: 1.9–2.9) in women and 2.2 mmol/L (Q1–Q3: 1.8–2.8) in men] (see Supplementary material online, 10). In 60.2% (95% CI: 60.0–60.4) of women and 60.9% (60.8–61.1) of men, LDL-c target levels were achieved within 18 months after initiation [median values: 2.2 mmol/L (Q1–Q3: 1.8–2.7) in women and 2.1 mmol/L (Q1–Q3: 1.7–2.6) in men]. About 36.8% (36.6–36.9) of women and 33.6% of men (33.4–33.7) achieved 50% of LDL lowering within 6 months of therapy and 42.1% (41.9–42.3) and 38.4% (38.3–38.6) achieved 50% of LDL lowering within 18 months of therapy.

**Figure 5:**
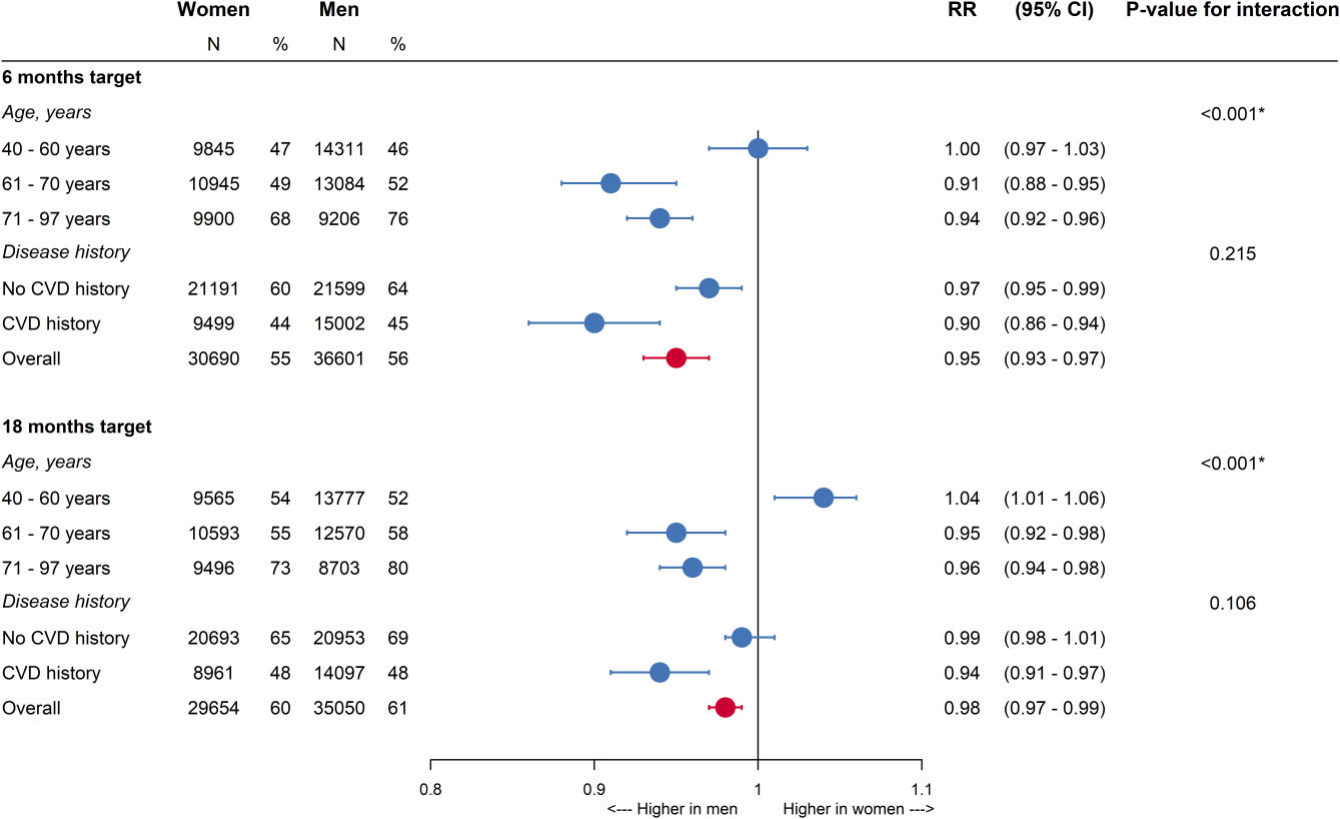
Women-to-men risk ratios for the achievement of LDL-c target levels. The main analyses were adjusted for age, SES, CVD history, diabetes, LDL-c levels, smoking status, BMI, year of initiation, statin intensity at initiation, use of blood pressure-lowering drugs, use of antiplatelets, and family history of IHD. The *N* refers to the number of women and men in each subgroup and the percentage refers to the percentage of women and men that attained LDL-c target levels in the 6 months and in the 18 months following statin initiation. RR: risk ratio and CI: confidence interval.

The adjusted women-to-men RR for achieving LDL-c target levels within 6 months was 0.95 (95% CI 0.93–0.97). The RR was 1.00 (0.97–1.03) in individuals aged 40–60 years, 0.91 (0.88–0.95) in individuals aged 61–70 years, and 0.94 (0.92–0.96) in individuals aged 71–97 years (*P*-value for interaction <0.001) (*Figure 5*). There was no statistically significant interaction between sex and CVD history (*P*-value: 0.215).

The women-to-men RR for achieving LDL-c target levels within 18 months after statin initiation was 0.98 (0.97–0.99). The RR was 1.04 (1.01–1.06) in individuals aged 40–60 years, 0.95 (0.92–0.98) in individuals aged 61–70 years, and 0.96 (0.94–0.98) in individuals aged 71–97 years (*P*-value for the interaction between sex and age <0.001). There was no interaction between sex and CVD history (*P*-value: 0.106) (*Figure 5*). The results of the unadjusted analyses are shown in Supplementary material online, 9.

The analyses with additional adjustment for the uptitration during follow-up showed similar results to the main analyses: RR 0.95 (0.93–0.97) and 0.98 (0.96–0.99) for target achievement within 6 months and 18 months, respectively.

## Discussion

This study examined sex differences in the occurrence of statin uptitration and LDL target achievement in a real-world setting. There are two main findings: first, among new statin users, women were less likely than men to be uptitrated for statin therapy, despite higher baseline cholesterol levels and after adjustment of individual characteristics and initial statin intensity. This finding was consistent in a subgroup of individuals that did not achieve LDL-c target levels within 6 months after statin initiation. Second, women were less likely to achieve target levels of LDL-c 6 months and 18 months after the initiation of statins.

### Sex differences in statin uptitration

Most studies on statin use have focused on prescriptions and dispensation rates, with few studies addressing statin uptitration. The finding in this study that women are less likely to be uptitrated than men aligns with previous evidence showing that women are prescribed statins of lower intensities than men.^6–8,10^ Other studies on statin uptitration have been conducted across different care settings,^18–20^ and have shown low rates of statin intensification. To our knowledge, only one other study investigated sex differences in statin uptitration in patients with diabetes in primary care and observed no sex difference.^21^ Our results show that individuals starting with a low-intensity statin were most likely to be uptitrated, with no observed sex differences. We also found that sex differences in statin uptitration were largest among those at younger age; where women were 31% less likely to be uptitrated as compared to men. This finding is in line with previous work suggesting that the largest sex differences in CVD prevention and management occur among younger individuals, which may be explained by the underestimation of the cardiovascular risk in women by both the practitioner and patient.^6,22^

### Achievement of LDL-c target levels

In this study, we found that younger women were more likely to reach the cholesterol targets compared to younger men. This can be explained by the existence of two cholesterol thresholds in the guidelines, which depends on age and CVD history. As CVD, on average, occurs later in life in women than men,^23^ women are more likely to have a target of 2.6 mmol/L (83% of women in the study vs. 72% of men), whereas more men will have a target of 1.8 mmol/L (17% of women vs. 28% of men). Our results on sex differences in target achievement did not differ when adjusting for the uptitration status at 6 months and we also found sex differences in uptitration in those not achieving the 6-month target level.

Guidelines recommend monitoring LDL-c as it serves as guide for clinical decisions in the potential intensification or modification of treatment.^16^ However, in our study, we observed a large proportion of missing data for the cholesterol measurements 6 months after statin initiation (i.e. 56% of women and 63% of men) and about one fourth of the study population (21% of women and 27% of men) had no LDL-c measurement recorded during follow-up.

### Strengths and limitations

The strengths of the study include the use of dispensing data from a large population reflecting routine clinical practice and providing insight in the complete patient journey and healthcare in the Netherlands. In addition, the new-user design reduced the risk of selection bias that might occur when including prevalent users to the study population. By ensuring that the individuals are new users of statins, we increased the comparability between women and men in terms of disease stage and prescribing behaviors. Also, the results of the sensitivity analysis on those without target achievement at 6 months strengthen the clinical relevance of the findings. The limitations of this study include the unavailability of tertiary care data, which might have captured additional data on medication use or LDL measurements. We have included immortal time bias in our analyses by including individuals with at least two dispensing of statins in our study population. However, this is unlikely to have change the sex differences as the criterium was required for both sexes and median times between the two first dispensing was only 21 days in women and 25 days in men. Missing data were common in this study, yet multiple imputation was used to retain statistical power and reduce bias. Since we only had data from 2010 onward and used baseline characteristics to define the target levels, some misclassification might have occurred. However, the misclassification is likely non-differential between sexes and is unlikely to have influenced the findings of the study. Though menopausal status is a predictor of blood cholesterol variation, it was not included in the analyses. However, all analyses were adjusted for age, and age has been shown a reasonable efficacy in predicting the onset of menopause.^24^ Cluster effects due to physician practice were not taken into account. PCSK9 inhibitors were not studied in this work as they were only recommended for the intensification of statins in the 2016 ESC guidelines and very few dispenses were available.^25^ Finally, adherence to therapy was not taken into account in the design of the study.

## Conclusions

To conclude, among new statin users, women are less likely to be uptitrated compared to men and to achieve cholesterol target levels. This study strengthens the evidence of the sex gap in statin treatment and reveals sex disparities throughout the course of therapy. Addressing these health inequities is key to ensure optimal and equitable cardiovascular treatment.

## Supplementary Material

qcaf017_Supplemental_File

## Data availability

Data are available upon reasonable request at the PHARMO Institute for Drug Outcome Research.
