# Supplementary material for: Uptitration of statin therapy in women and men: a population-based cohort study
Source: Eur Heart J Qual Care Clin Outcomes. 2025 Apr 17;11(8):1310–8. doi: 10.1093/ehjqcco/qcaf017 (PMC12714376; doi:10.1093/ehjqcco/qcaf017)
Supplement: qcaf017_Supplemental_File [file qcaf017_supplemental_file.docx]

**Supplementary Material 1**. Flowchart of the study population.


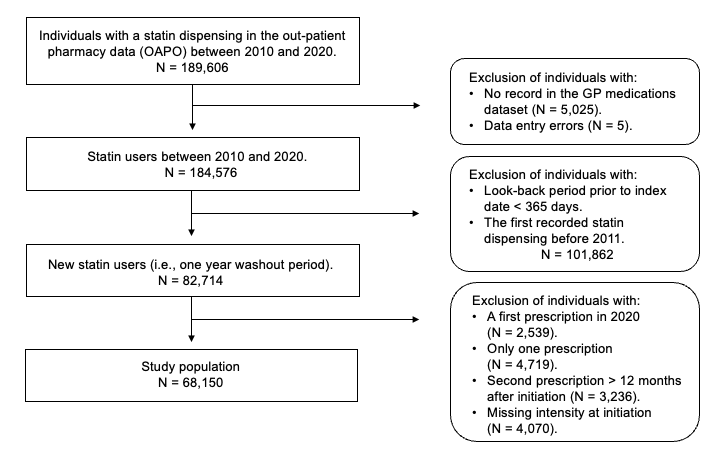


**Supplementary Material 2**. REPEAT-IT Graphical depiction of the study design.

**Main analyses (a)**

**
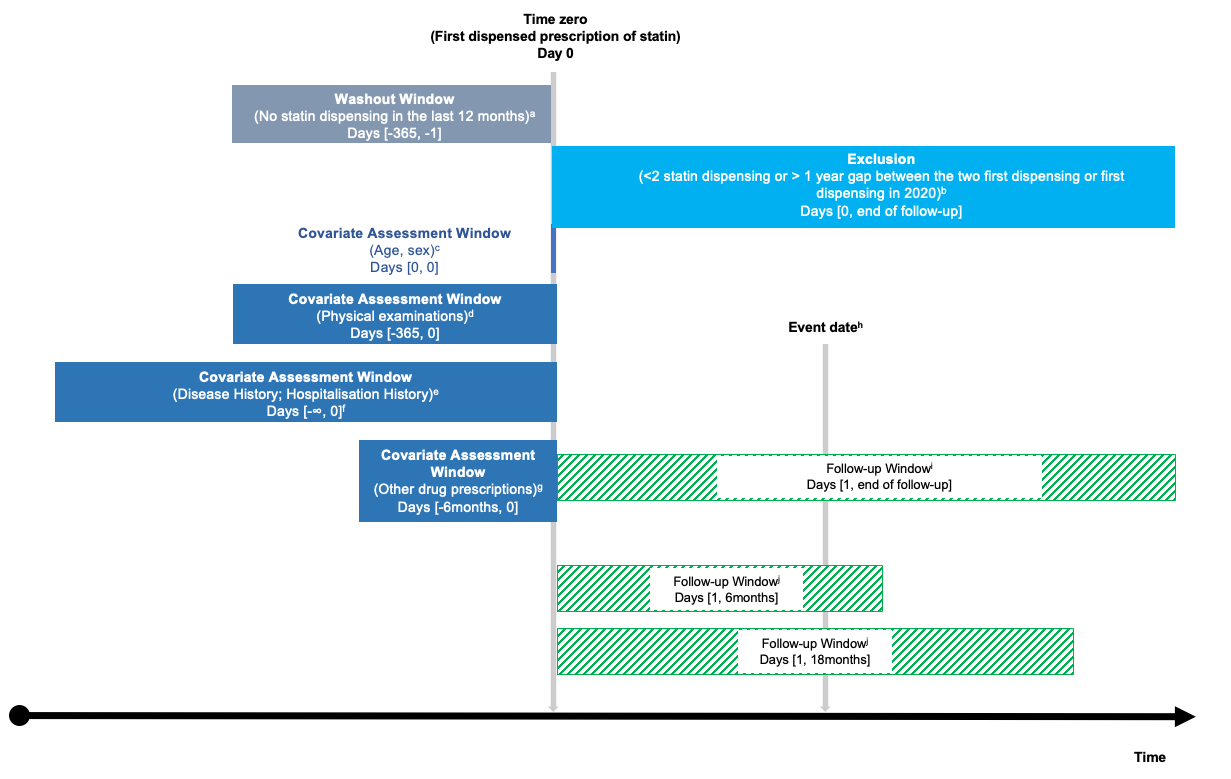
**

1. Individuals were included if they had at least one dispensed statin prescription between January 2011 and December 2020, had at least one year of database history at the index date and no previous statin dispensing in the year before the index date.
2. Individuals were excluded from the study population if they had less than two statin dispenses between January 2011 and December 2020, with a maximum one-year gap between the first two dispenses, or their first dispensing in 2020.
3. Baseline characteristics at index date: age, sex, socio-economic status and intensity of the first dispensed statin.
4. Physical examinations recorded within a year before the index date were extracted: systolic blood pressure (SBP), diastolic blood pressure (DBP), body mass index (BMI), weight, height, glucose, smoking, total cholesterol (TC), LDL-c, high density lipoprotein (HDL-c), triglycerides (TG), average glucose level (HbA1c), hemoglobin, estimated glomerular filtration rate (eGFR). Smoking status at index date was retrieved from the medical records.
5. History of CVD, coronary heart disease, stroke, heart failure, diabetes and family history of ischemic heart disease were extracted from the medical records and were coded using The International Classification of Primary Care (ICPC) and The International Classification of Diseases – 10 (ICD-10). See Supplementary Material 3.
6. The data are available from January 2010 onwards.
7. Data on use of antihypertensive medication and antiplatelet medication was extracted from the out-patient pharmacies database and use was defined as at least one recorded dispensed prescription in the six months before the index date.
8. Outcome: first uptitration of statin therapy, achievement of LDL-c target levels.
9. Earliest of: outcome of interest, death, moving out of the data source or end of follow-up.
10. Follow-up windows of 6 months and 18 months for the achievement of LDL-c target levels.

**Analysis on the subset of the study population that didn’t achieve cholesterol targets within the first six months (b)**

**
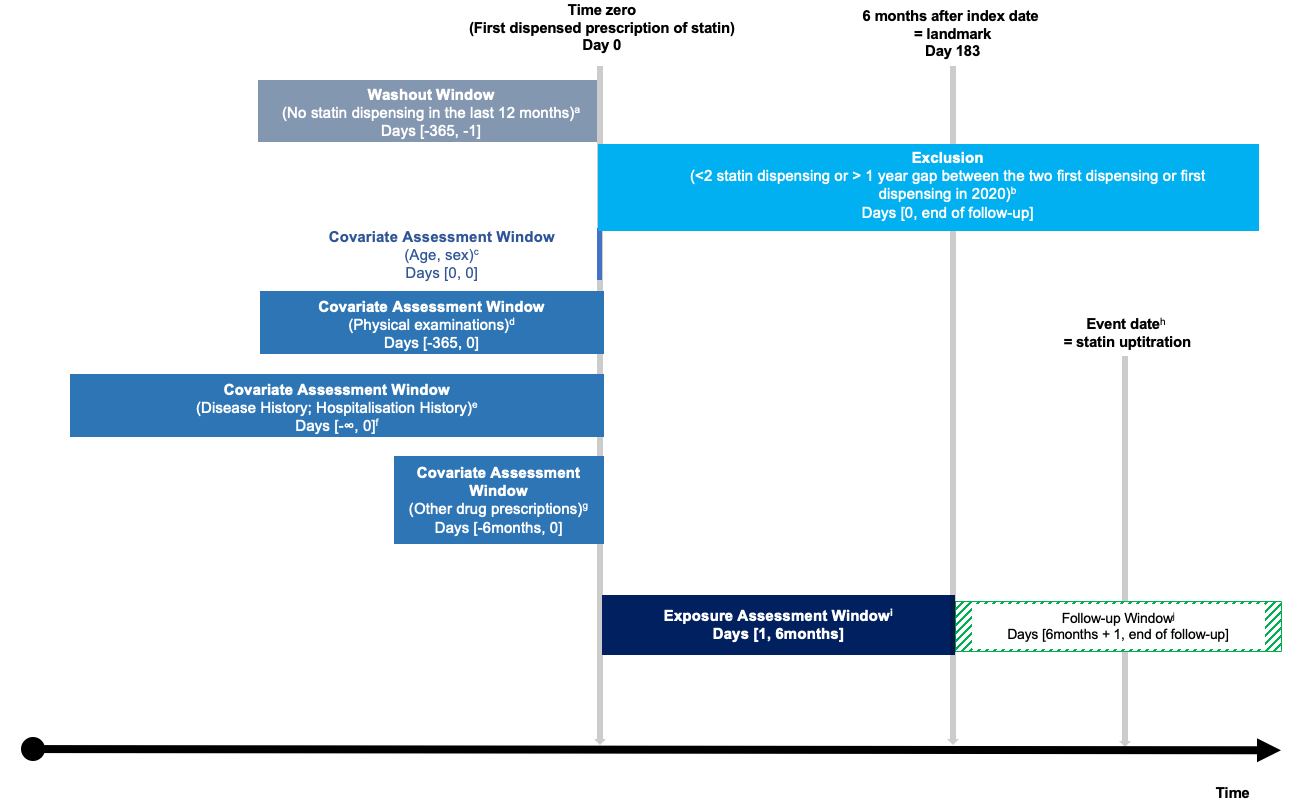
**

1. Individuals were included if they had at least one dispensed statin prescription between January 2011 and December 2020, had at least one year of database history at the index date and no previous statin dispensing in the year before the index date.
2. Individuals were excluded from the study population if they had less than two statin dispenses between January 2011 and December 2020, with a maximum one-year gap between the first two dispenses, or their first dispensing in 2020.
3. Baseline characteristics at index date: age, sex, socio-economic status and intensity of the first dispensed statin.
4. Physical examinations recorded within a year before the index date were extracted: systolic blood pressure (SBP), diastolic blood pressure (DBP), body mass index (BMI), weight, height, glucose, smoking, total cholesterol (TC), LDL-c, high density lipoprotein (HDL-c), triglycerides (TG), average glucose level (HbA1c), hemoglobin, estimated glomerular filtration rate (eGFR). Smoking status at index date was retrieved from the medical records.
5. History of CVD, coronary heart disease, stroke, heart failure, diabetes and family history of ischemic heart were extracted from the medical records and were coded using The International Classification of Primary Care (ICPC) and The International Classification of Diseases – 10 (ICD-10). See Supplementary Material 3.
6. The data are available from January 2010 onwards.
7. Data on use of antihypertensive medication and antiplatelet medication was extracted from the out-patient pharmacies database and use was defined as at least one recorded dispensed prescription in the six months before the index date.
8. Outcome: first uptitration of statin therapy.
9. Exclusion of individuals if achievement of LDL-c target levels, occurrence of statin uptitration or death within the first six months.
10. Earliest of: outcome of interest, death, moving out of the data source or end of follow-up.

**Supplementary Material 3**. Definitions and codes for medical history and drug prescriptions.

|  |  |
| --- | --- |
| Definition of cardiovascular history | |
| *ICPC codes* | |
| K74 | Angina pectoris |
| K7401 | Unstable angina pectoris |
| K7402 | Stable angina pectoris |
| K75 | Acute myocardial infarction |
| K76 | Other/chronic ischemic heart disease |
| K7601 | Coronary sclerosis |
| K7602 | Myocardial infarction (>4 weeks ago) |
| K77 | Heart failure |
| K78 | Atrial fibrillation |
| K7902 | Ventricular tachycardia |
| K83 | Non-rheumatic valve disease |
| K8402 | Atrioventricular block |
| K8403 | Cardiomyopathy |
| K89 | Transient ischemic attack |
| K90 | Cerebrovascular accident |
| K9001 | Subarachnoid hemorrhage |
| K9002 | Intracerebral hemorrhage |
| K9003 | Stroke / cerebrovascular accident |
| K9201 | Peripheral artery disease |
| K9901 | Aortic aneurysm |
| *ICD10 codes* | |
| I* | All codes for diseases of the circulatory system |
| J81 | Pulmonary edema |
| K761 | Other diseases of liver |
| G45 | Transient cerebral ischemic attacks and related syndromes |
| R57 | Shock |
| *ICD9 codes* |  |
| 410 | Acute myocardial infarction |
| 411 | Other acute and subacute forms of ischemic heart disease |
| 412 | Old myocardial infarction |
| 413 | Angina pectoris |
| 414 | Coronary atherosclerosis |
| 415 | Acute pulmonary heart disease |
| 430 | Subarachnoid hemorrhage |
| 431 | Intracerebral hemorrhage |
| 432 | Other intracranial hemorrhage |
| 433 | Occlusion and stenosis of precerebral arteries |
| 434 | Occlusion of cerebral arteries |
|  |  |
| ICPC codes for stroke |  |
| K89 | Transient ischemic attack |
| K90 | Cerebrovascular accident |
| K9001 | Subarachnoid hemorrhage |
| K9002 | Intracerebral hemorrhage |
| K9003 | Stroke / cerebrovascular accident |
|  |  |
| ICPC codes for heart failure |  |
| K77 | Heart failure |
|  |  |
| ICPC codes for diabetes | |
| T90 | Diabetes mellitus |
| T90.01 | Diabetes mellitus type 1 |
| T90.02 | Diabetes mellitus type 2 |
|  |  |
| ICD10 codes for family history of IHD |  |
| Z824 | Family history of ischemic heart disease and other diseases of the  circulatory system |
| ATC codes of the different statin types | |
| C10AA01 | Simvastatin |
| C10AA03 | Pravastatin |
| C10AA04 | Fluvastatin |
| C10AA05 | Atorvastatin |
| C10AA07 | Rosuvastatin |
| C10AA08 | Pitavastatin |
|  |  |
| ATC codes for the definition of antihypertensive medications | |
| C02* | Antihypertensive |
| C03* | Diuretics |
| C07* | Beta blockers |
| C08* | Calcium channel blockers |
| C09A*/C09B* | ACE-i |
| C09C*/C09D* | ARB |
|  |  |
| ATC codes for the definition of antiplatelets medications | |
| B01AC* | Platelet aggregation inhibitors excl. heparin |
|  |  |
|  |  |

With ICPC = International Classification of Primary Care ; ICD = International Classification of Diseases; IHD = Ischemic Heart Disease ; ATC = anatomical therapeutic chemical classification.

* Refers to: ‘starting with’.

**Supplementary Material 4**. Statin Intensities following the ACC/AHA guidelines*

| High-Intensity Statin Therapy | Moderate-Intensity Statin Therapy | Low-Intensity Statin Therapy |
| --- | --- | --- |
| Daily dose lowers LDL-C, on average, by approximately ≥50% | Daily dose lowers LDL-C, on average, by approximately 30% to <50% | Daily dose lowers LDL-C, on average, by <30% |
| - Atorvastatin 40-80 mg - Rosuvastatin 20-40 mg | - Atorvastatin 10-20 mg - Rosuvastatin 5-10 mg - Simvastatin 20-40 mg - Pravastatin 40-80 mg - Lovastatin 40 mg - Fluvastatin XL 80 mg - Fluvastatin 40 mg BID - Pitavastatin 2–4 mg | - Simvastatin 10 mg - Pravastatin 10-20 mg - Lovastatin 20 mg - Fluvastatin 20-40 mg |

With LDL-c = low-density lipoprotein cholesterol
Reference: Stone NJ, Robinson JG, Lichtenstein AH, Bairey Merz CN, Blum CB, Eckel RH, et al. 2013 ACC/AHA Guideline on the Treatment of Blood Cholesterol to Reduce Atherosclerotic Cardiovascular Risk in Adults. Circulation. 2014 Jun 24;129(25_suppl_2):S1–45.

**Supplementary Material 5**. Definition of uptitration.

1. Creation of a score per dispense.

| Condition | Score (created for each dispensing = row) |
| --- | --- |
| If the intensity is low (*for definitions, see Supplementary Material 4*) | 1 |
| If the intensity is moderate | 2 |
| If the intensity is high | 3 |
| If the dispensing is ezetimibe | 1 |
| If the dispensing is a drug-combination and the intensity of the statin part is moderate | 3 |
| If the dispensing is a drug-combination and the intensity of the statin part is high | 4 |
| If intensity is missing | *na* |

1. The scores were summed per day (NB: This step was done to take into account the occurrence of multiple dispenses on the same day (e.g., one statin and one ezetimibe)).
2. Creation of *uptitration* for each day per individual.

| Condition | Uptitration |
| --- | --- |
| If score > baseline score | 1 |
| If score <= baseline score | 0 |
| If score is missing | *na* |

**Supplementary Material 6**. Model specification for the multiple imputation.

| Variables used as predictors in the multiple imputation (stratified by sex) | | Imputed |
| --- | --- | --- |
| General characteristics |  |  |
| Age | - Continuous - Categorical (40-60;60-70;70-99 years old) | No |
| Socio-economic status | - Categorical (1;2;3) | Yes |
| Smoking | - Categorical (yes;previously;never) | Yes |
| Deceased | - Categorical (yes;no) | No |
| Clinical characteristics |  |  |
| Systolic blood pressure | - Continuous | Yes |
| Diastolic blood pressure | - Continuous | Yes |
| Glucose | - Continuous | Yes |
| Creatinine | - Continuous | Yes |
| eGFR | - Continuous | Yes |
| LDL-c | - Continuous | Yes |
| Total cholesterol | - Continuous | Yes |
| Triglycerides | - Continuous | Yes |
| BMI | - Continuous | Yes |
| Target levels related |  |  |
| LDL-c 6 months | - Continuous | Yes |
| LDL-c 18 months | - Continuous | Yes |
| Disease history |  |  |
| CVD | - Categorical (yes;no) | No |
| CHD | - Categorical (yes;no) | No |
| Stroke | - Categorical (yes;no) | No |
| Diabetes | - Categorical (yes;no) | No |
| Heart failure | - Categorical (yes;no) | No |
| Family history of IHD | - Categorical (yes;no) | No |
| Medication use |  |  |
| ATC code | - Categorical (C10AA01;C10AA03;C10AA04; C10AA05;C10AA07) | No |
| Year of first prescription | - Continuous | No |
| Number of dispensed items | - Continuous | Yes |
| Intensity of statin at initiation | - Categorical (1;2;3) | No |
| Previous use of antihypertensives | - Categorical (yes;no) | No |
| Previous use of antiplatelets | - Categorical (yes;no) | No |
| Uptitration related | | |
| Uptitration | - Binary | Yes |
| Days to uptitration | - Continuous | Yes |

**Supplementary Material 7**. Uptitration patterns in the study population.

1. **Overall**

| **Intensity at start*** | **Uptitration** | **Low** | **Low + E** | **Moderate** | **Moderate + E** | **High** | **High + E** | **Combo Moderate** | **Combo High** |
| --- | --- | --- | --- | --- | --- | --- | --- | --- | --- |
| Low | 1159 (12) |  | 34 (3) | 1040 (90) | 9 (1) | 63 (5) | 1 (0) | 8 (1) | 1 (0) |
| Moderate | 7986 (83) |  |  |  | 504 (6) | 6890 (86) | 62 (1) | 407 (5) | 60 (1) |
| High | 482 (5) |  |  |  |  |  | 423 (88) |  | 42 (10) |

1. **Women**

| **Intensity at start*** | **Uptitration** | **Low** | **Low + E** | **Moderate** | **Moderate + E** | **High** | **High + E** | **Combo Moderate** | **Combo High** |
| --- | --- | --- | --- | --- | --- | --- | --- | --- | --- |
| Low | 644 (17) |  | 15 (2) | 590 (92) | 5 (1) | 31 (5) | 0 (0) | 1 (0) | 1 (0) |
| Moderate | 3149 (80) |  |  |  | 182 (6) | 2757 (88) | 16 (1) | 140 (4) | 20 (1) |
| High | 133 (3) |  |  |  |  |  | 116 (87) |  | 11 (9) |

1. **Men**

| **Intensity at start*** | **Uptitration** | **Low** | **Low + E** | **Moderate** | **Moderate + E** | **High** | **High + E** | **Combo Moderate** | **Combo High** |
| --- | --- | --- | --- | --- | --- | --- | --- | --- | --- |
| Low | 515 (9) |  | 19 (4) | 450 (87) | 4 (1) | 32 (6) | 1 (0) | 7 (1) | 0 (0) |
| Moderate | 4837 (85) |  |  |  | 322 (7) | 4133 (85) | 46 (1) | 267 (6) | 40 (1) |
| High | 349 (6) |  |  |  |  |  | 307 (88) |  | 34 (10) |

NB: The table shows the patterns of uptitration among uptitrated individuals. The brackets show the percentages. Some numbers don’t add up to 100% in the table. This is due to the occurrence of multiple dispenses of the same intensity on the same day, which was defined as uptitration following the scoring system (Suppl. Material 5), but that don’t fit with the logic of this table.

*Some individuals started therapy with ezetimibe already but since the numbers were very small, there were added to the broader categories.

**Supplementary Material 8**. Results of the Cox regression for the uptitration analysis, stratified by age and statin intensity.

| Model | | **N** | | **HR** | **95% CI** |
| --- | --- | --- | --- | --- | --- |
| Age category (years) | **Statin intensity** | **N women** | **N men** |  |  |
|  |  |  |  |  |  |
| 40-60 | Low | 465 | 383 | 0.97 | (0.79-1.18) |
| 40-60 | Moderate | 8665 | 12137 | **0.66** | **(0.62-0.71)** |
| 40-60 | High | 825 | 1936 | **0.68** | **(0.50-0.92)** |
| 61-70 | Low | 492 | 411 | 0.93 | (0.75-1.15) |
| 61-70 | Moderate | 9729 | 11138 | **0.72** | **(0.66-0.78)** |
| 61-70 | High | 831 | 1682 | **0.70** | **(0.49-0.98)** |
| 71-97 | Low | 485 | 347 | 0.88 | (0.68-1.15) |
| 71-97 | Moderate | 8754 | 7788 | **0.73** | **(0.65-0.83)** |
| 71-97 | High | 839 | 1243 | 0.86 | (0.48-1.53) |
|  |  |  |  |  |  |

The analyses were adjusted for age, socio-economic status, cardiovascular disease history, diabetes, family history of ischemic heart disease, low-density lipoprotein cholesterol levels, smoking status, body mass index, year of initiation, statin intensity at initiation, use of blood pressure-lowering drugs, use of antiplatelets. The N refer to the number of women and men in each subgroup. HR = hazard ratio; CI = confidence interval.

**Supplementary Material 9**. Women-to-men unadjusted and adjusted estimates the analyses.

|  |  |  |  |  |
| --- | --- | --- | --- | --- |
| Uptitration | |  |  |  |
| In the entire study population (N = 68,150) | | HR unadjusted | 0.81 | (0.77-0.84) |
|  |  | HR adjusted | 0.72 | (0.69-0.75) |
| Among individuals that didn’t achieve LDL-c targets  in the first 6 months (N = 25,922) | | HR unadjusted | 0.80 | (0.76-0.84) |
|  |  | HR adjusted | 0.72 | (0.68-0.76) |
|  | |  |  |  |
| LDL-c target levels achievement | |  |  |  |
| Within 6 months (N = 67,291) | | RR unadjusted | 0.98 | (0.96-1.00) |
|  |  | RR adjusted | 0.95 | (0.93-0.97) |
| Within 18 months (N = 64,704) | | RR unadjusted | 0.99 | (0.98-1.00) |
|  |  | RR adjusted | 0.98 | (0.97-0.99) |

The analyses were adjusted for age, socio-economic status, cardiovascular disease history, diabetes, family history of ischemic heart disease, low-density lipoprotein cholesterol levels (LDL-c), smoking status, body mass index, year of initiation, statin intensity at initiation, use of blood pressure-lowering drugs, use of antiplatelets. The N refer to the number of women and men in each subgroup. HR = hazard ratio; RR = risk ration; CI = confidence interval.

**Supplementary Material 10**. Distribution of LDL-c in women and men six months after statin initiation.


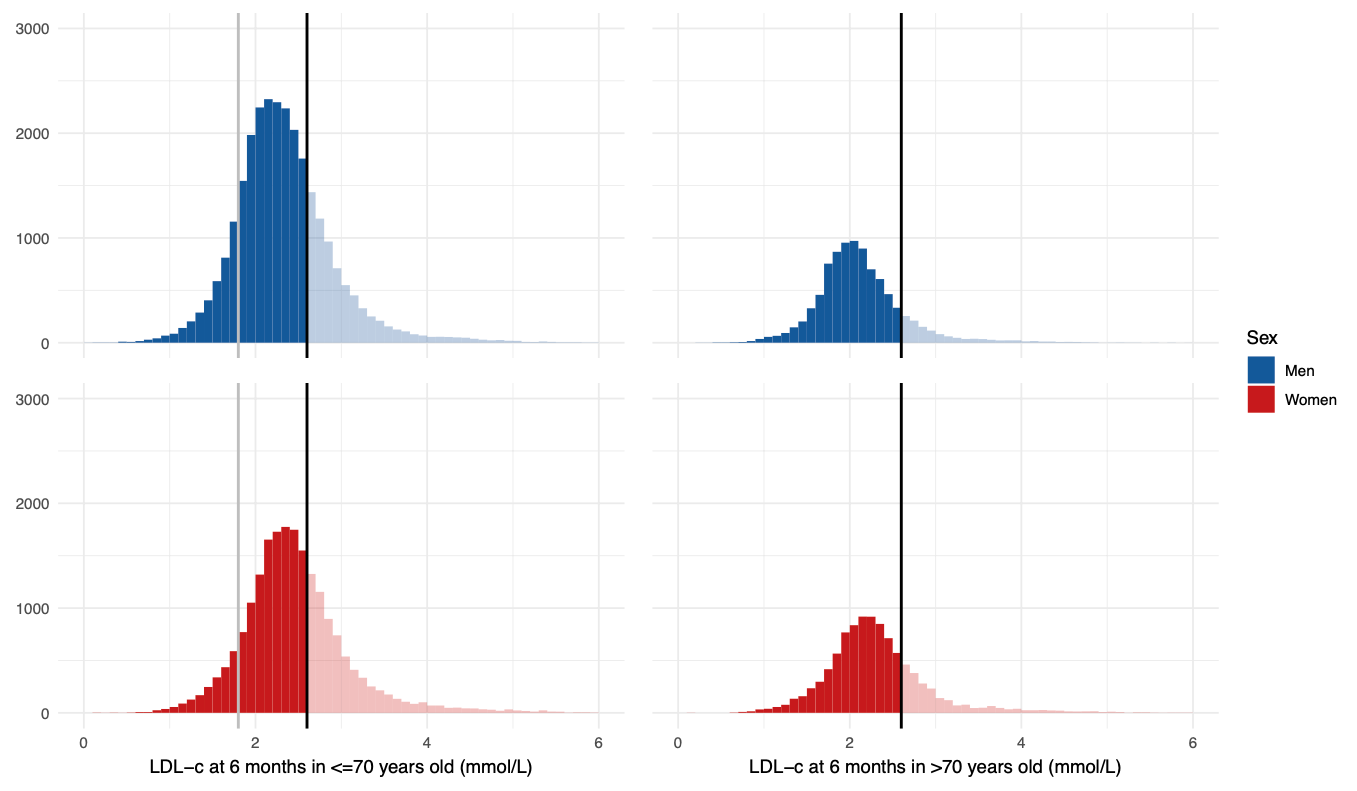


With LDL-c = low-density lipoprotein cholesterol. The y axis indicates the counts. The grey and black lines show the guidelines-recommended targets of 1.8mmol/L and 2.6mmol/L.
